# Benefits of intraoperative endoscopy: case report and review of 300 sleeves gastrectomies

**DOI:** 10.1186/s13022-015-0023-0

**Published:** 2015-12-19

**Authors:** Ameer Gomberawalla, Rami Lutfi

**Affiliations:** Department of Surgery, Presence Saint Joseph Hospital, 2900 N Lake Shore Drive, Chicago, IL 60657 USA; Chicago Institute of Advanced Bariatrics, Chicago, IL USA

**Keywords:** Sleeve gastrectomy, Bariatric surgery, Endoscopy

## Abstract

**Background:**

The laparoscopic sleeve gastrectomy (LSG) is the fastest rising bariatric procedure being performed in the United States. Some surgeons advocate for an intra-operative endoscopy for their leak test, while others utilize air via a form of an oral gastric tube. We present a case demonstrating the benefits of endoscopy intra-operatively as well as discuss our experience of 200 consecutive sleeve gastrectomies.

**Case presentation:**

The case is a 37 years old female undergoing LSG for treatment for morbid obesity. As is our practice, we routinely perform an intra-operative endoscopy to help ensure a tight seal on the remnant stomach prior to completion of the procedure. During our endoscopy, a blood clot overlying a portion of the esophagus was noted, evacuated and evaluated and found to have a non-bleeding mucosal tear. This was unusual in our experience. There was no leak found on intra-operative endoscopy. After the patient woke up, she starting to have bouts of hematemesis. The decision was made to go back to the OR and evaluate her endoscopically. rather than attempting a laparoscopy based off of our endoscopic findings. Upon take back, we found a mucosal tear in her distal esophagus that now started bleeding, and her staple line was intact. Hemostasis was successfully achieved with two epinephrine injections. The remaining portion of her postoperative course was uncomplicated.

**Discussion and conclusions:**

The patient was able to be safely managed with a post-operative EGD. The intra-operative endoscopic findings allowed us to be more confident that this was an esophageal issue rather than a staple line problem, and were able to start with EGD prior to laparoscopy. Additionally, from a visualization perspective, the intra-operative endoscopy allows you to fully visualize the staple line, evaluate for twists or narrowing, and test for leak with confidence. From a residency standpoint, it also increases the confidence of residence to perform endoscopy on intubated patients.

## Background

The laparoscopic sleeve gastrectomy (LSG) has become increasingly popular in the field of bariatric surgery, comprising 36.3 % of all bariatric surgeries performed in academic centers, and an even higher percentage in community based hospitals, and is predicted to become the most popular form of bariatric surgery [1, 2]. The optimal construction technique and many other technical aspects are still under debate by experts. Amongst these topics is the routine use of an intraoperative leak test. After construction of the sleeve, the vast majority of surgeons will perform some form of a leak test. We believe that the use of intraoperative endoscopy is the optimal form of sleeve inspection after construction and leak test. Our paper describes our experience with a case where the information gathered from the endoscopy was utilized immediately for the patient’s benefit. We also describe our experience with over 300 sleeve gastrectomies utilizing intraoperative endoscopy.

## Case presentation

The patient is a 37 year old female with a past medical history significant for morbid obesity, gastroesophageal reflux disease, and asthma. Her past surgical history is significant for a previous tubal ligation and laparoscopic cholecystectomy. Her preoperative BMI was 47.6. She chose to undergo a LSG after her previous attempts at weight loss were unsuccessful. The patient was taken into operating room and we initiated our procedure with a 5 mm incision in the left upper midclavicular line and inserted a trochar under direct vision with a 5 mm 0° camera. We placed four working ports total, as well as a Nathanson liver retractor. The sleeve technique involves freeing the greater curvature starting 5 cm proximal to the pylorus, with complete dissection to the left crus to avoid leaving any retained fundus behind. This patient also had a small 1 cm hiatal hernia which was dissected and then closed with an anterior figure of eight stitch. The sleeve was constructed over a 34 French Ewald tube with a reinforced staple line (SEMGUARD, Gore Arizona), though we often use a blunt tip bougie. Special attention is paid to avoid tightness at the incisura and angle of His. After creation of the sleeve, which was uneventful, the patient had her routine post-operative endoscopy, in which a blood clot was noticed in the lower esophagus. This was an unusual finding during our endoscopies. *There was not a significant amount of esophagitis noted on the EGD.* Once in the stomach, the staple line was found to be straight and there was no bubbling on our leak test and no blood in the stomach or staple line. The patient was then extubated after the specimen was removed and brought to the post-anesthesia care unit (PACU).

**Fig. 1:**
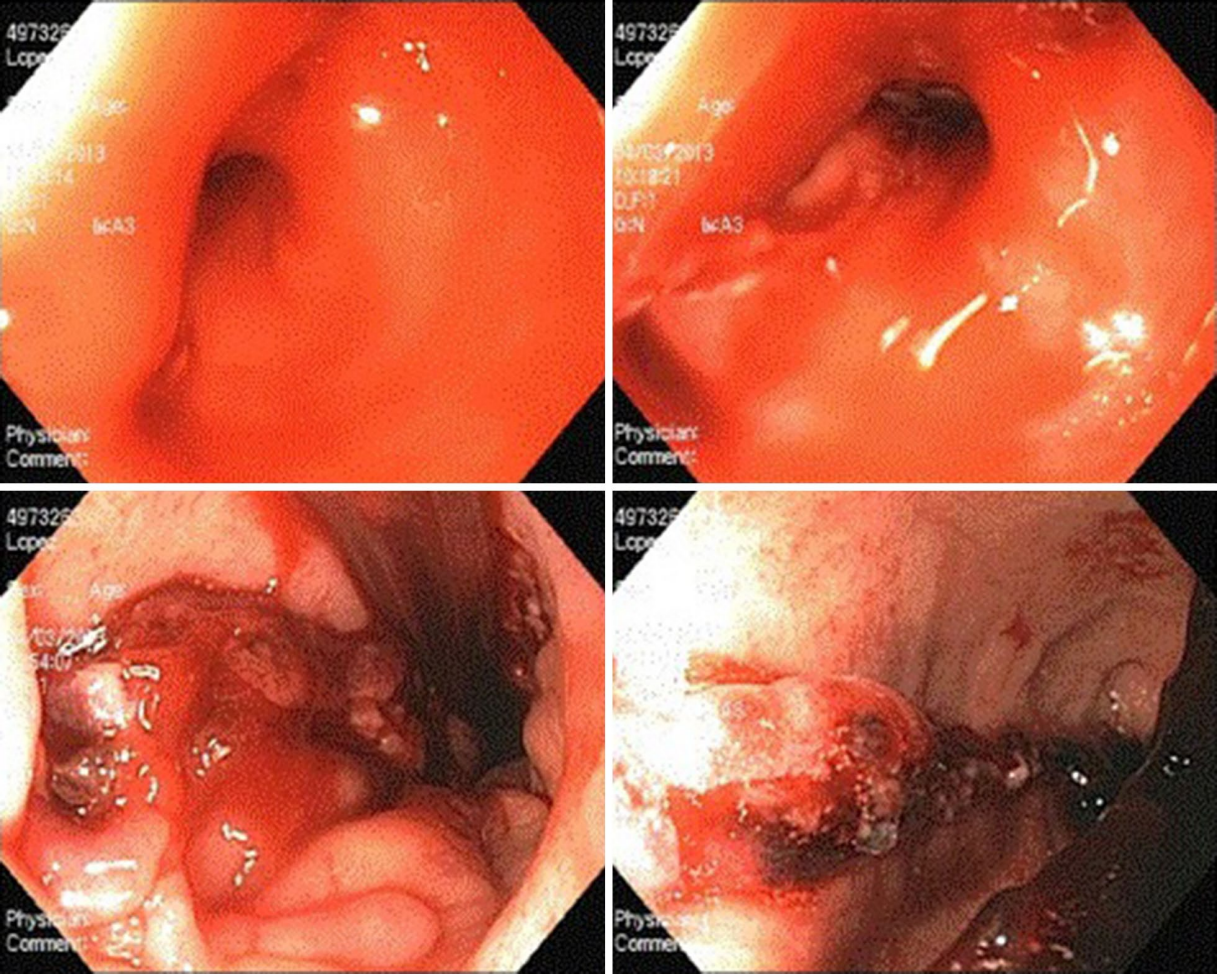
Endoscopic findings

**Fig. 2:**
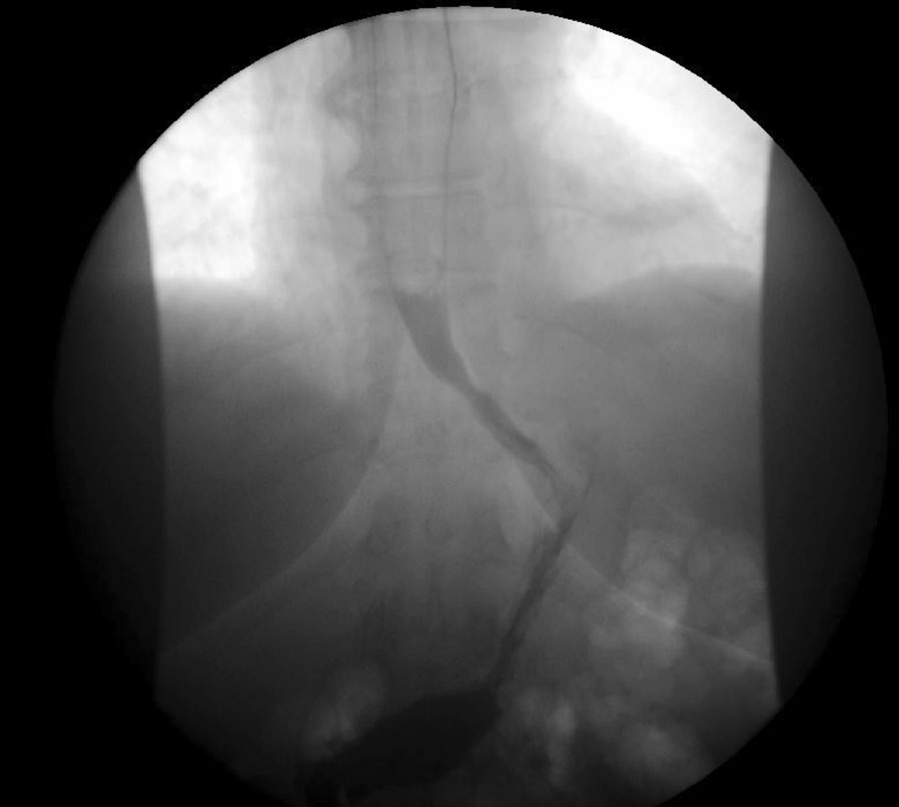
Patient’s upper GI POD #1

In the PACU, about 15 min after extubation, the patient started to develop hematemesis, about 10 cc every 2–3 min of fresh blood. We remembered our unusual finding of blood in the lower esophagus and reintubated the patient to bring her back into the operating room for an endoscopy. The findings are demonstrated in Fig. 1. There was a small tear in the lower esophagus that was bleeding. This area was injected with 7 ml of 1:10,000 epinephrine. The bleeding ceased at this point. The staple line was in tact. The abdominal cavity was not entered in the operating room as we felt that there were no areas of concern in the stomach during our initial endoscopy and this remained the case upon second inspection. Under direct vision, an orogastric tube was placed. The patient was kept intubated for 6 h post-operatively and then extubated without difficulty. She was monitored in the intensive care unit for the first night. She remained hemodynamically stable with stable hemoglobins of 11.0–13.0 g/dl. The remainder of her post-operative course was unremarkable. We routinely perform an upper GI on post operative day 1 to assess for leak or obstruction. Hers is shown in Fig. 2. Her postoperative visits have all gone well and she has had no long term sequelae of this event. She followed up with us at 6 months and she has had 53 % excess weight loss with no dysphagia or reflux symptoms.

## Discussion

As the LSG gains momentum as a primary operation for morbid obesity, many technical aspects are areas of debate. These can include the size of the bougie utilized, the beginning of the distal section, reinforcement of staple line, and seal verification [3]. The purpose of this report is to discuss our experience with utilizing intraoperative endoscopy as our primary seal verification, and to inspect the newly created staple line for bleeding or twists. Data from our experience is represented in Table 1. We have had only 1 leak in over 300 cases, far below most published cohorts [4].

**Table 1:** Patient characteristics

	Average	Range
Age (years)	43	(19–72)
BMI (kg/m^2^)	48	(33–78)
Mean OT (min)	101	(37–272)
EWL 6 months (%BW)	54.9	(19–102)
EWL 12 months (%BW)	70.2	(20–133)

The use of a leak test is a common practice in bariatric surgery. For the sleeve gastrectomy, people use air, methylene blue, or no test at all. Only two reports describe the use of intraoperative endoscopy thus far. Diamentis et al. [5] reports their experience with 25 patients. The mean operative time was 117 min, longer than our time. They demonstrated no leaks intraoperatively. A novel approach is also discussed by Frezza and colleagues [6], who discuss using a 29 F endoscope that serves as both the bougie and then it is already in place for a leak test.

There are multiple benefits to performing intraoperative endoscopy. The first is demonstrated by this case, allowing for identification of bleeding that may direct management strategies down the road. If we did not see the blood clot initially, it is likely we would have initially started laparoscopically, or at least checked the staple line from the intra-abdominal viewpoint at some time during the re-operation. The ability to check for internal bleeding within the staple line also gives additional assurance and information to the surgeon. The bougies that are placed are not benign instruments, and can cause damage to either the stomach or esophagus upon insertion. Checking the lumen after sleeve creation allows for early identification of potential injury. There has also been discussion in the literature regarding the use of pre-operative endoscopy to rule out certain disease processes that may affect creation of the sleeve or affect the bariatric procedure in question for primary bariatric procedures. In general, prospective studies that implemented pre-operative endoscopy demonstrated changes the surgical treatment in about 2–3 % of cases. Additionally, the information obtained did alter the medical management for GERD in approximately 50 % [7, 8]. In our case, it is possible that a pre-operative endoscopy would have revealed a specific pathology such as a stricture that could have led to a subsequent injury, but the lack of symptoms suggests that this is just speculative. The injury more likely represents the risks of a Bougie/Ewald tube being used, and intra-operative endoscopy allowed for identification of this. For revisional bariatric surgery, we and other authors agree that pre operative endoscopy is essential to evaluate causes of failure of the initial procedure [9].

The second main benefit is of technical skill and education. This is a great opportunity for the surgeon to gain expertise in endoscopy, and become familiar with maneuvering through a sleeve. Initial intubation of the esophagus can be challenging as you do not have the aid of the patient’s swallowing reflex. Additionally, as endoscopic treatments for operative complications are becoming more prevalent, the surgeon must be aware of the appearance of normal sleeve construction. From a resident/teaching hospital standpoint, this is an excellent opportunity for the residents to enhance their skills and increase their endoscopy cases. In a time where some residencies may struggle to to meet this requirement, these cases give residents additional opportunities without adding significant time to their training. As the implementation of the fundamentals of endoscopic skills (FES) module starts with the intern class of 2014, we have further impetus to as many endoscopic modalities to the training of residents as we can [10]. Further studies will shine light as to whether a resident’s volume of sleeve gastrectomies using intraoperative endoscopy has any effects on either initial FES scores or final FES scores.

## Conclusion

Overall, our experience has been very positive with utilization of intraoperative endoscopy after creation of our sleeve gastrectomy. Our leak rate is extremely low and occurred very early in our experience, and we believe that taking the extra time to perform a leak test using an endoscope will pay dividends for both the practitioner and the patient.

## Abbreviations

POD: post-operative day
UGI: upper gastro-intestinal imaging
FES: fundamentals of endoscopic skills
LSG: laparoscopic sleeve gastrectomy
ICU: intensive care unit
PACU: post-anesthesia care unit
